# Cold Exposure-Induced Up-Regulation of Hsp70 Positively Regulates PEDV mRNA Synthesis and Protein Expression In Vitro

**DOI:** 10.3390/pathogens9040246

**Published:** 2020-03-26

**Authors:** Fanzhi Kong, Yaru Xu, Wei Ran, Baishuang Yin, Li Feng, Dongbo Sun

**Affiliations:** 1 State Key Laboratory of Veterinary Biotechnology, Harbin Veterinary Research Institute, Chinese Academy of Agricultural Sciences, Harbin 150069, China; fanzhikong110@hotmail.com; 2 Laboratory for the Prevention and Control of Swine Infectious Diseases, College of Animal Science and Veterinary Medicine, Heilongjiang Bayi Agricultural University, No. 5 Xinfeng Road, Sartu District, Daqing 163319, China; yaruxu_byau@126.com (Y.X.); weiranpid@126.com (W.R.); 3 College of Animal Science and Technology, Jilin Agricultural Science and Technology University, 132101 Jilin, China; ybs3421@126.com

**Keywords:** cold exposure, Hsp70, porcine epidemic diarrhea virus, viral mRNA synthesis and protein expression

## Abstract

Porcine epidemic diarrhea (PED) is a highly contagious, intestinal infectious disease caused by porcine epidemic diarrhea virus (PEDV). PEDV as an emerging and re-emerging epizootic virus of swine causes substantial economic losses to the pig industry in China and other countries. In China, the occurrence of PED shows significant seasonal variations, usually outbreak during the winter season. The epidemic characteristics of PED may be highly correlated with the changes of ambient temperature. However, molecular mechanism on the seasonal occurrence of PED still remains unclear. It has been widely observed that low ambient temperature up-regulates the expression of host heat shock protein 70 (Hsp70). Here, we showed that nucleotide and protein levels of Hsp70 were up-regulated in the intestinal of cold exposed pig and cold exposed Vero E6 cells. We found that overexpression of Hsp70 could increase PEDV mRNA synthesis and protein expression in Vero E6 and IPEC-J2 cells, while the siRNAs mediated knockdown of Hsp70 and VER155008 mediated inhibition of Hsp70 resulted in inhibition of viral mRNA synthesis and protein expression in Vero E6 cells. These data suggested that Hsp70 positively regulated PEDV mRNA synthesis and protein expression, which being helpful for understanding the seasonality of PED epidemics and development of novel antiviral therapies in the future.

## 1. Introduction

Porcine epidemic diarrhea virus (PEDV), belonging to the order *Nidovirales* subfamily of *Coronaviridae*, which causes porcine epidemic diarrhea (PED), an enteric disease characterized by acute watery diarrhea, dehydration, vomiting, and high mortality in nursery piglets [1]. In the winter of 2010, PED outbreaks occurred in China and cause large economic losses [2]. Subsequently, in spring of 2013, first PED was reported in US, which lead to astonishing mortality in winter of 2013/2014, severely affect the pork industry [3,4]. PEDV outbreaks frequently occur during early spring and winter months in temperate regions, peaking between November and March [5,6,7,8]. Cold stress of piglets caused by fluctuations in temperature is thought to contribute to the seasonality of PEDV, while the significance of cold stress in PEDV infection is still unclear.

Heat shock proteins (Hsps), molecular chaperones originally discovered based on their up-regulation in response to heat stress. Evidence is growing that exposure of cells and tissues to low ambient temperature leads to transcription and translation of heat shock proteins 70 KDa (Hsp70) [9,10,11,12,13,14]. Moreover, Hsp70 has been shown to be one key factor for multiple virus infection including rabies virus [15], porcine circovirus type 2 [16], porcine reproductive and respiratory syndrome virus [17], influenza A virus [18], dengue virus [19], infectious bronchitis virus [20], Epstein-Barr virus [21], ectromelia virus [22], hepatitis B virus [23], enterovirus A71 [24] and zika virus [25,26]. Hsp70 plays important role in many steps of viral life cycle, including entry [27,28], uncoating [29], replication of the viral genome [30,31], gene expression [32] and virion morphogenesis [33,34]. However, the function of Hsp70 in PEDV replication has not been investigated, which may explain the seasonality of PED epidemics.

In this study, Hsp70 expression in the intestinal of cold exposed pig and cold exposed Vero E6 cells were investigated. The role of Hsp70 in PEDV replication has also been studied in Vero E6 and IPEC-J2 cells. We showed that cold exposure could induce Hsp70 expression levels in vivo and in vitro. Also, Hsp70 levels were in parallel with the viral mRNA synthesis and protein expression levels in vitro. These results indicated that exposure to low ambient temperature may enhance PEDV replication through up-regulated Hsp70 in the intestinal which may contribute to the seasonality of PED epidemics, suggesting environmental temperature control is important to prevent PEDV infection and disease outcomes in the cold seasons.

## 2. Materials and Methods 

### 2.1. Virus, Reagents and Antibodies

PEDV strain CV777 (GenBank accession no. KT323979; group I) and polyclonal antibody against PEDV N protein were kindly provided by the Division of Swine Digestive System Infectious Diseases, State Key Laboratory of Veterinary Biotechnology, Harbin Veterinary Research Institute, Chinese Academy of Agricultural Sciences. For virus propagation in Vero E6 cells, the maintenance medium was DMEM supplement with 1% penicillin/streptomycin and 10 μg/mL trypsin (2.5% Trypsin, Gibco). Small interfering RNA targeting Hsp70 (siRNA-Hsp70) and nonspecific siRNA (siRNA-NC) were designed and synthesized by GenePharma (Suzhou, China). Hsp70 inhibitor VER155008 (SML0271) was obtained from Sigma. The mouse anti-Hsp70 monoclonal antibody (mAb) (ab5439) was purchased from abcam (Shanghai, China), and the mouse anti-GAPDH mAb (bsm-33033M), goat anti-mouse IgG/HRP were purchased from Bioss (Shanghai, China).

### 2.2. Cold Exposure of Piglets and Sample Collection

To investigate the effects of cold exposure on Hsp70 expression in pig duodenum, jejunum, and ileum, nine piglets with 35 days of age from the same birthday were randomly allocated to three groups as one control and two cold exposure (n = 3/each group) groups. Animals were allowed to acclimate for three days before being subjected to experimental conditions. Prior to cold exposure, pigs were raised in a climatic chamber at an ambient temperature of 26 ± 2 °C with 40% relative humidity. Experimental pigs were exposed to cold ambient temperature at 4 °C for 6 h and 12 h, while control pigs were exposed to room temperature (26 ± 2 °C) for 12 h. At indicated time points, piglets were euthanized and duodenum, jejunum and ileum were collected immediately then frozen in liquid nitrogen and stored in −80 °C freezer until use. All animal studies were approved in accordance with the regulations and guidelines of the animal care committee of Heilongjiang Bayi Agricultural University (20180328002), and all experiments were performed in accordance with specific guidelines provided by Heilongjiang Bayi Agricultural University (201401002).

### 2.3. Cold Exposure of Vero E6 Cells and Sample Collection

To investigate the effects of cold exposure on Hsp70 expression in African green monkey kidney epithelial (Vero E6) cells, three groups of cell as one control (37 °C) and two cold exposure (4 °C or 25 °C) groups were prepared. Vero E6 cells were grown in Dulbecco’s modified Eagle medium (DMEM) supplemented with 10% fetal bovine serum (FBS) and 1% penicillin/streptomycin. When monolayer reach 80%–90% confluent, culture medium was replaced and exposed to 4 or 25 °C for 6 or 12 h respectively, followed by recovery at 37 °C for another 4 h. Control cells were cultured at 37 °C all through the experiments. At the indicated time points, cells were collected after three times of washing. Proteins and RNA were extracted according to manufacturer’s protocol.

### 2.4. Quantitative RT-PCR

Total RNA was extracted from the tissue or cellular samples using RNA extraction kit from Tiangen Biotech (Beijing, China). Reverse transcription was carried out using PrimeScript RT Master Mix (Takala, Dalian, China) according to the manufacturer’s instructions. Two microliter of the RT reaction mixture was submitted to quantitative RT-PCR (qRT-PCR) using gene specific primers (Table 1) and NovoStar^®^SYBR qPCR SuperMix Plus (E096-01B, Novoprotein, Shanghai, China), according to the manufacturer’s recommendations. The reaction procedure was 95 °C for 2 min, followed by 40 cycles at 95 °C for 15 s and 60 °C for 60 s. The relative amount of target gene mRNA was normalized to that of β-actin mRNA in the same sample. To confirm specific amplification, melting curve analysis of the RT-PCR products was performed according to the manufacturer’s protocol. The qRT-PCR was performed in an ABI QuantStudio 3 Real-Time PCR system. Relative quantification analysis was performed using the cycle threshold (2^−ΔΔCt^) method [35].

### 2.5. Western Blotting

Equivalent amounts of tissue or cellular lysate proteins were subjected to 12% sodium dodecyl sulfate-polyacrylamide gel electrophoresis and transferred to polyvinylidene difluoride membranes (0.45 μm). After blocking with 5% (*w*/*v*) non-fat milk, membranes were incubated at 37 °C for 1 h with mouse monoclonal or polyclonal antibodies against GAPDH, Hsp70 and PEDV N protein. After three times washing with 0.05 % Tween-20 in tris-buffered saline (TBST), the membranes were incubated at 37 °C for 1 h with horseradish peroxidase (HRP)-conjugated goat anti-mouse IgG. Protein detection was performed using LuminataTMCrescendo Western HRP substrate (Merck KGaA, Darmstadt, Germany) and Amersham Imager 600 (GE Healthcare, Chicago, IL, USA). Protein levels were determined using ImageJ quantification software (National Institutes of Health, Bethesda, MD, USA). 

### 2.6. Overexpression of Hsp70 by Eukaryotic Expression Vector

To investigate the effects of Hsp70 on PEDV replication, plasmid encoding porcine Hsp70 was transfected into Vero E6 and porcine small intestinal epithelial cells (IPEC-J2). To construct the recombinant overexpression vector, porcine Hsp70 (HSPA6, reference sequence: NM_001123127) coding sequence without the stop codon was cloned from the total cDNA of porcine kidney (PK-15) cells. The Hsp70 fragment was amplified by PCR and inserted into the multiple cloning site of the pTSB plasmid (TSE02, TranSheepBio, Shanghai, China). The recombinant pTSB-Hsp70 vectors were successfully constructed, as confirmed by DNA sequencing analysis (data not shown). To determine the effects of Hsp70 on PEDV replication, Vero E6, and IPEC-J2 cells plated on 6-well plates were transient transfected with 2.5 μg/well pTSB-Hsp70 plasmid using Exfect 2000 transfection reagent according to the manufacturer’s recommendations (Vazyme, Nanjing, China). IPEC-J2 cells were purchased from Ze Ye (Shanghai, China) and cultured in Roswell Park Memorial Institute (RPMI) 1640 medium supplement with 10% FBS and 1% penicillin/streptomycin. At 24 h post-transfection, cells were infected with PEDV strain CV777 at a multiplicity of infection (MOI) of 1. At 48 h post inoculation (hpi), samples were subjected to detection of PEDV RNA and protein by qRT-PCR and immunoblotting, respectively.

### 2.7. Knockdown of Hsp70 by Silencing RNA

To investigate the effects of silenced monkey Hsp70 on PEDV replication, three small interference RNAs (siRNAs) targeting the monkey Hsp70 (siRNA-Hsp70-1, 5′-GGAGGUAUCCUCUAUGGUUTT-3′; siRNA-Hsp70-2, 5′-GCAGAAAGAAACGUGCUUATT-3′; siRNA-Hsp70-3, 5′-CCUGAUGAAGCUGUUGCUUTT-3′) were synthesized. Non-specific siRNA (siRNA-NC, 5′-UUCUCCGAACGUGUCACGUTT-3′) was used as control. Vero E6 cells grown to approximately 30%–50% confluence in 6-well plates were transfected with siRNA-Hsp70 (40 nmol/well) or siRNA-NC (40 nmol/well), respectively, using Exfect 2000 transfection reagent (Vazyme, Nanjing, China) according to the manufacturer’s instruction. Twenty-four hours post transfection; cells were washed three times with PBS, and infected with PEDV strain CV777 at 1 MOI. At 24 hpi, samples were subjected to detection of PEDV RNA and protein by qRT-PCR and immunoblotting, respectively.

### 2.8. Cell Viability Assay

To determine the effects of Hsp70 inhibitor VER155008 on Vero E6 cell viability, MTT (3-(4,5-dimethylthiazol-2-yl)-2,5-diphenyltetrazolium bromide) assay was applied. Briefly, Vero E6 cells were grown on 96-well culture plates at a density of 5 × 10^3^/well and were incubated for 24 h. After initial period of incubation, cells were treated with various concentrations of VER155008 for another 24 h. Cell viability was assessed using the MTT Cell Cytotoxicity Assay Kit (Beyotime, Beijing, China) as the manufacturer’s recommendations.

### 2.9. Time-of-addition Assay

To validate the effects of VER155008 on PEDV replication, Vero E6 cells were seeded onto 6 well plates at a density of 5 × 10^5^/well in complete culture medium incubated for 24 h. Then, cells were inoculated with PEDV strain CV777 (MOI = 1) for 2 h at 4 °C to synchronize infection. After three wash with PBS, 1 mL of DMEM contain trypsin (10 μg/mL) was added to each well and placed in a humidified incubator at 37°C. At the indicated time points, a VER155008 solution was added to the wells at a final concentration of 5 μM. At 24 hpi all cells were harvest for immunoblotting assays. Time of VER155008 additions are shown in Figure 4A.

### 2.10. Statistical Analysis

Statistical analysis was performed using GraphPad PRIM software (version 8.2.0 for Windows; GraphPad Software Inc., San Diego CA, USA). Data were analyzed to establish their significance using one-way analysis of variance (ANOVA), followed by the least-significant difference test, and expressed as means ± SD. Differences were regarded as significant at *P* < 0.0001 (****), *P* < 0.001 (***), *P* < 0.01 (**) or *P* < 0.05 (*). 

## 3. Results

### 3.1. Cold Exposure Increases Hsp70 Expression In Vivo and In Vitro

**Figure 1:**
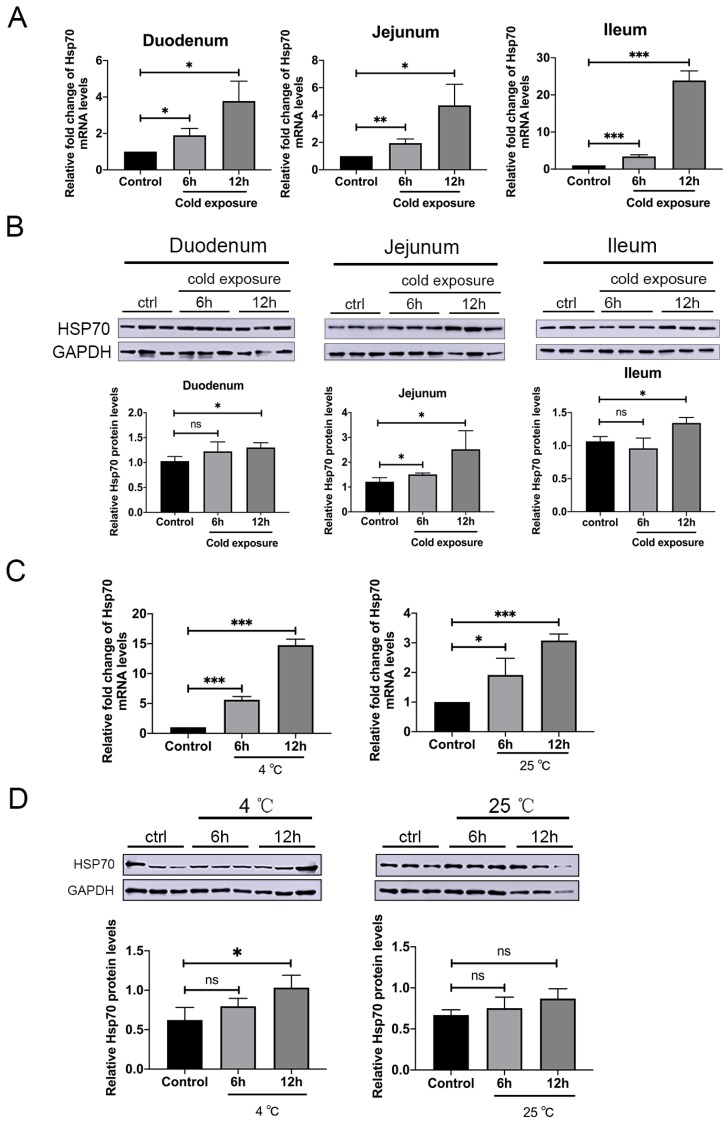
Cold exposure increases the expression of Hsp70 in vivo and in vitro. (**A**) Relative fold change of Hsp70 mRNA levels in vivo were analysed by qRT-PCR. The Hsp70 mRNA levels were normalized to the level of β-actin mRNA in the same sample. (**B**) Duodenum, jejunum and ileum from control or cold exposed piglets were lysed and extracts were analyzed by western blotting using anti-Hsp70, or anti-GAPDH antibodies. The level of relative proteins was quantified by immunoblot scanning and normalized with respect to the amount of GAPDH (lower panel). (**C**) Relative fold change of Hsp70 mRNA levels in vitro were analysed by qRT-PCR. The Hsp70 mRNA levels were normalized to the level of β-actin mRNA in the same sample. (**D**) Control or cold exposed cells were lysed and extracts were analyzed by western blotting using anti-Hsp70, or anti-GAPDH antibodies. The level of relative proteins was quantified by immunoblot scanning and normalized with respect to the amount of GAPDH (lower panel). The data are presented as the mean ± SEM (n = 3). Statistically significant differences are indicated as follows: ns, not significant; *, *P* < 0.05; **, *P* < 0.01; ***, *P* < 0.001.

We have investigated the effect of cold exposure on the expression of Hsp70 in vivo and in vitro. Duodenum, jejunum, and ileum of cold exposed piglets were analyzed at different time point by qRT-PCR and western blotting. The Hsp70 expression was enhanced in the duodenum, jejunum, and ileum after 12 h of cold exposure (Figure 1A,B). Especially in the jejunum, the most strongly tissue tropism of PEDV, Hsp70 protein levels were up-regulated after 6 h of cold exposure (Figure 1B). Similarly, nucleic acid and protein levels of Hsp70 were also increased in 4 °C exposed Vero E6 cells, while under 25 °C exposure, only nucleic acid levels of Hsp70 increased (Figure 1C,D). These data indicated that Hsp70 expression levels could be up-regulated by the cold exposure in vivo and in vitro.

### 3.2. Overexpression of Hsp70 Enhance PEDV mRNA Synthesis and Protein Expression In Vero E6 and IPEC-J2 Cells

**Figure 2:**
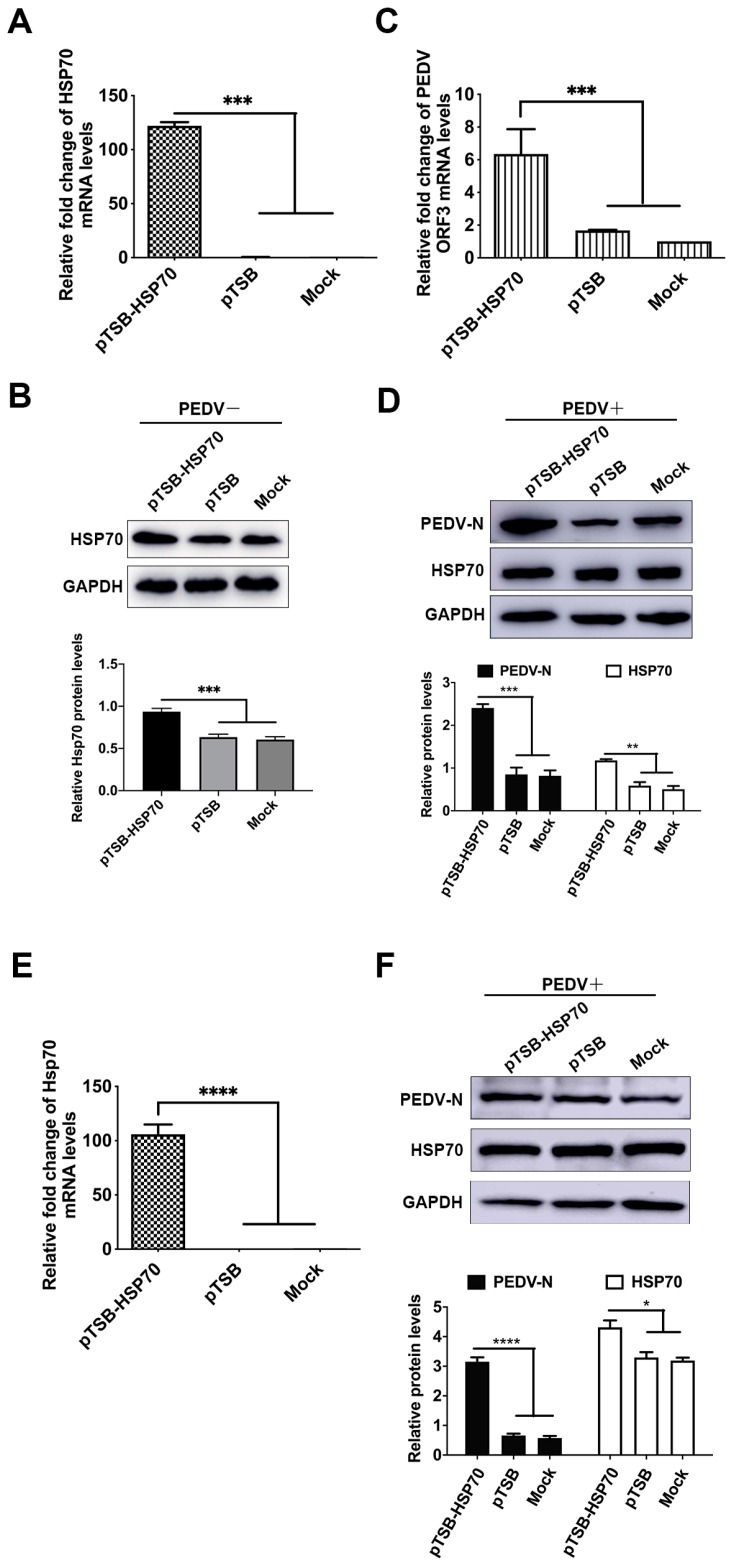
Overexpression of Hsp70 promotes PEDV mRNA synthesis and protein expression. (**A**) Plasmid coding porcine Hsp70 were transfected into Vero E6 cells, and incubated for 72 h. The Hsp70 mRNA levels were detected by qRT-PCR. The level of Hsp70 mRNA was normalized to the level of β-actin mRNA in the same sample. (**B**) The cells were harvested for western blotting analysis with anti-Hsp70 anti-GAPDH antibodies. The levels of Hsp70 was quantified by immunoblot scanning and normalized with respect to the amount of GAPDH (lower panel). (**C**) Plasmid coding porcine Hsp70 were transfected into Vero E6 cells, 24 h post transfection PEDV were infected at MOI = 1 and incubated for another 48 h. The Hsp70 and PEDV ORF3 mRNA levels were detected by qRT-PCR. The mRNA levels of Hsp70 and PEDV ORF3 were normalized to the level of β-actin mRNA in the same sample. (**D**) The cells were harvested for western blotting analysis with anti-Hsp70, anti-PEDV-N and anti-GAPDH antibodies. The relative protein levels were quantified by immunoblot scanning and normalized with respect to the amount of GAPDH (lower panel). (**E**) Plasmid coding porcine Hsp70 were transfected into IEPC-J2 cells, 24h post transfection PEDV were infected at MOI = 1 and incubated for another 48 h. The Hsp70 mRNA levels were detected by qRT-PCR. The level of Hsp70 mRNA was normalized to the level of β-actin mRNA in the same sample. (**F**) The cells were harvested for western blotting analysis with anti-Hsp70, anti-PEDV-N and anti-GAPDH antibodies. The relative protein levels were quantified by immunoblot scanning and normalized with respect to the amount of GAPDH (lower panel). Statistically significant differences are indicated as follows: ns, not significant; *, *P* < 0.05; **, *P* < 0.01; ***, *P* < 0.001; ****, *P* < 0.0001.

To address our hypothesis that overexpression of Hsp70 could enhance PEDV replication, we used eukaryotic expression vector to overexpress porcine Hsp70 into Vero E6 and IPEC-J2 cells. As shown in Figure 2A,B, pTSB-Hsp70 successfully transduced an exogenous gene and expressed the target protein in Vero E6 cells. When Hsp70 was overexpressed by pTSB-Hsp70, an increase in PEDV mRNA and N protein levels were observed compare to both pTSB and mock groups (Figure 2C,D). As shown in Figure 2E,F, overexpression of Hsp70 can also enhance PEDV N protein levels in IPEC-J2 cells. These data suggested that overexpression of Hsp70 can significantly enhance PEDV mRNA synthesis and protein expression in Vero E6 and IPEC-J2 cells.

### 3.3. Silencing of Hsp70 by siRNA inhibit PEDV mRNA Synthesis and Protein Expression in Vero E6 Cells

**Figure 3:**
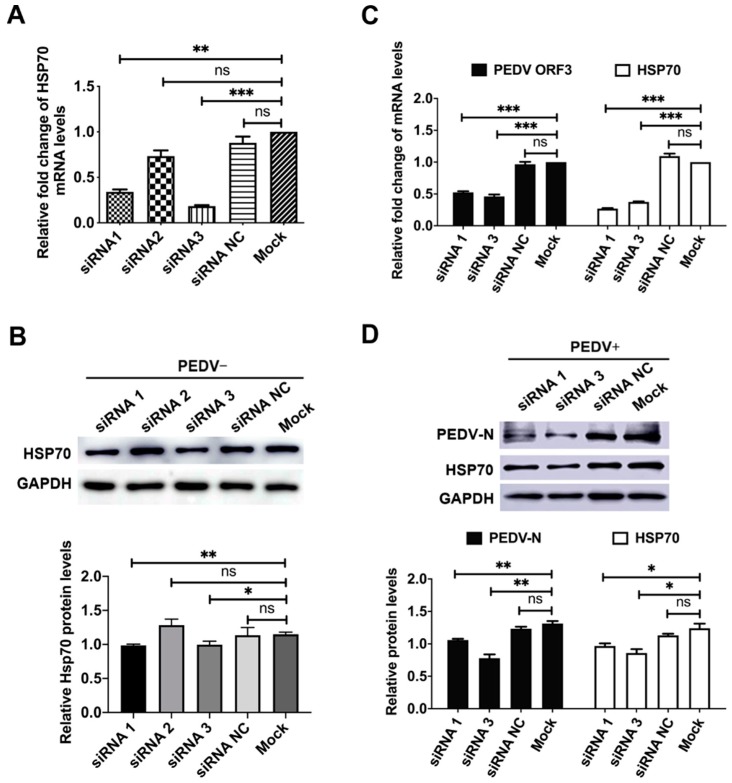
Knockdown of Hsp70 reduced PEDV mRNA synthesis and protein expression. (**A**) Vero E6 cells were transfected with siRNA1, siRNA2, siRNA3 and nonspecific control (siRNA NC) targeting the Hsp70 gene, and incubated for 48 h. The Hsp70 mRNA levels were detected by qRT-PCR. The level of Hsp70 mRNA was normalized to the level of β-actin mRNA in the same sample. (**B**) The cells were harvested for western blotting analysis with anti-Hsp70 anti-GAPDH antibodies. The levels of Hsp70 was quantified by immunoblot scanning and normalized with respect to the amount of GAPDH (lower panel). (**C**) Vero E6 cells were transfected with siRNA1, siRNA3 and siRNA NC targeting the Hsp70 gene, 24 h post transfection PEDV were infected at MOI = 1 and incubated for another 24 h. The Hsp70 and PEDV ORF3 mRNA levels were detected by qRT-PCR. (**D**) The cells were harvested for western blotting analysis with anti-Hsp70, anti-PEDV-N and anti-GAPDH antibodies. The relative protein levels were quantified by immunoblot scanning and normalized with respect to the amount of GAPDH (lower panel). The data are presented as the mean ± SEM (n = 3). Statistically significant differences are indicated as follows: ns, not significant; *, *P* < 0.05; **, *P* < 0.01; ***, *P* < 0.001.

We performed silencing experiments to investigate more specifically the function of Hsp70 expression in PEDV replication. We designed three siRNAs, siRNA1, siRNA2, siRNA3, and a negative control siRNA (siRNA NC) targeting the Hsp70 gene. SiRNA2 had no inhibited effect, while siRNA 1 and siRNA3 had significantly inhibited Hsp70 mRNA and protein levels compared to scramble siRNA (Figure 3A,B). SiRNA1 and siRNA3 were used to investigate the relationship between Hsp70 synthesis and PEDV replication. Twenty-four hours post siRNAs transfection, cells were infected with PEDV at 1 MOI. Under these conditions, the knockdown of Hsp70 expression was associated with a significant reduction of the amounts of viral mRNA and protein levels (Figure 3C,D). These results confirmed that the specific inhibition of Hsp70 synthesis led to a reduction of viral RNA and protein levels, suggesting Hsp70 was involved in the viral transcription and translation during PEDV replication.

### 3.4. VER155008 Inhibit PEDV N Protein Expression by Inhibit Virus Invasion and Replication Phase

**Figure 4:**
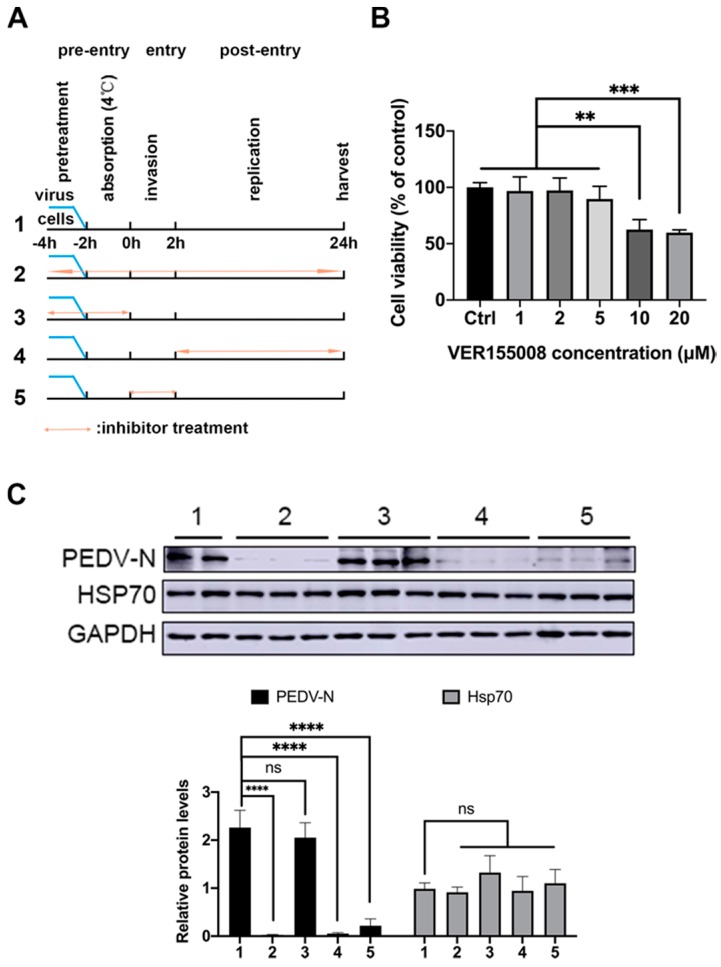
Hsp70 inhibition blocks PEDV invasion and replication. (**A**) Timeline of VER155008 addition. (**B**) Vero E6 cells were treated with serial concentrations of VER1555008 as indicated for 24 h. Cell viability was measured by MTT assay. DMSO treated cells served as control (Ctrl). (**C**) Vero E6 cells were treated by VER155008 at different phases and harvest at 24h post infection. Cell extracts were analyzed by western blotting with anti-Hsp70, anti-PEDV-N and anti-GAPDH antibodies. The relative protein levels were quantified by immunoblot scanning and normalized with respect to the amount of GAPDH (lower panel). The data are presented as the mean ± SEM (n = 3). Statistically significant differences are indicated as follows: ns, not significant; **, *P* < 0.01; ***, *P* < 0.001; ****, *P* < 0.0001.

To further investigate the stage at which Hsp70 exerts its pro-viral effect, VER155008 was added to the PEDV infected Vero E6 cells at different time points before and during infection. The infection process was divided into virus absorption (at 4 °C), virus invasion (the first two hours post infection at 37 °C) and replication (2–24 hpi) phases (Figure 4A). Cytotoxicity of VER155008 on Vero E6 cells was measured by MTT assay after 24 h incubation, results suggest that application of 5 μM VER155008 causing no cell toxicity by comparison to the control cells (Figure 4B). Twenty-four hours post infection, samples were harvested and analyzed to measure levels of viral protein. Compare to the control group (group 1), VER155008 inhibited PEDV N protein levels significantly if applied throughout the experiment (group 2) or during the replication (group 4) and invasion stages (group 5), although no inhibition of PEDV production were shown in group 3 (Figure 4C). These data indicated that inhibition of Hsp70 by VER155008 seriously impaired the production of PEDV by inhibit viral invasion and replication.

## 4. Discussion

Seasonality of the PED epidemic may link to environmental factors such as the drop of ambient temperature. Physiological stress experienced under cold exposure leads to a weakening of immune response [36] and increased Hsp70 levels [13,14]. It has been reported that Hsp70 could promote influenza A virus replication through modulates polymerase activity [18]. Also, low ambient temperature has been shown to shape the seasonality of influenza [37,38]. Obviously, low environmental temperature is related to the outbreak and spread of infectious diseases.

In this study, we tried to determine whether low environmental temperature induced Hsp70 impact on PEDV infection by using piglets, Vero E6 and IPEC-J2 cells. Thermal sensitivity and grade of physiological stress were defined according to Predicted Mean Vote (PMV) and Physiologic Equivalent Temperature (PET), 4 °C was the critical temperature of strong cold stress and extreme cold stress [39]. Meanwhile, the average temperature in the cold region of northeast China was during −5 °C to 5 °C. So we choose 4 °C for cold exposure experiment in this study. Exposure of piglets to low ambient temperature increased Hsp70 levels significantly in the duodenum, jejunum, and ileum, where tropism of PEDV infection in vivo.

Similarly, exposure of cultured Vero E6 cells to low ambient temperature also increased Hsp70 mRNA and protein levels. Although only mRNA levels of Hsp70 were significantly up regulated when Vero E6 cells were exposed to 25 °C environment, we still notice the increase tendency of Hsp70 protein levels as cold exposure time extension, suggesting Hsp70 protein synthesis rate were correlated with cell stress levels. Overexpression of Hsp70 in Vero E6 and IPEC-J2 cells or siRNAs mediated knockdown of Hsp70 in Vero E6 cells resulted in increased or decreased mRNA synthesis and protein expression of PEDV respectively. Specific inhibition of Hsp70 by VER155008 seriously impaired the N protein expression of PEDV by inhibits viral invasion and replication. Our data suggest that cold exposure induced Hsp70 could contribute to the infection of PEDV and the seasonality of PED epidemics.

The heat shock response or stress response is the most highly conserved cellular response in all species. Hsps play important roles in antigen presentation, intracellular trafficking, apoptosis, and act as molecular chaperones by helping nascent polypeptides assume their proper conformations [40]. The increase of Hsp70 levels following physical stress has been widely observed. The induction may occur indirectly through the production of a large number of unfolded proteins during stress. Indeed, Hsp70 is involved in many cellular processes, such as cell cycle, apoptosis, and cellular innate immunity pathways, which is frequently diverted by viruses for their benefits [11,41,42,43]. Induction of the Hsp70 is a specific virus function that ensures proper synthesis of viral proteins and virions, thus stress proteins may be important for virus replication [44]. To clarify whether Hsp70 could be induced under cold exposure in vivo and in vitro, pigs and cultured cells were exposed to cold ambient temperature for indicated time and found that Hsp70 was up-regulated (Figure 1). However, the mechanism of increased expression of Hsp70 under cold exposure is unknown and consequently need to be further investigated.

Viral infection depends entirely on the host cellular factors at different steps of the viral life cycle: viral invasion, genome replication, viral protein synthesis, viral assembly, and counter defense against cell apoptosis and innate immunity. It has been shown that Hsp70 is involved in all steps of viral life cycle for viruses from numerous families of diverse orders [45,46]. To determine the relationship between Hsp70 levels and PEDV replication, pTSB-Hsp70 mediated overexpression or siRNAs mediated knockdown of Hsp70 were applied in PEDV infected Vero E6 or IPEC-J2 cells, results indicate that Hsp70 has a pro-viral effect on PEDV mRNA synthesis and protein expression by acting during at least one step of the viral cycle (Figure 2 and Figure 3). To further clarify which steps in the viral life cycle require Hsp70 in Vero E6 cells, we performed time-of-addition experiment using VER155008. As expected, PEDV N protein levels were significantly reduced when VER155008 applied throughout the experiment (Figure 4, group 2). Interestingly, PEDV N protein levels were also inhibited significantly if applied VER155008 during the replication (Figure 4, group 4) and invasion stages (Figure 4, group 5). In contrast, VER155008 had no effects on PEDV N protein levels if applied during absorption stage (Figure 4, group 3). Not surprisingly, Hsp70 levels were not change during VER155008 treatment (Figure 4C). VER155008 is a chemical modulator that interfere with Hsp70 ATPase enzymatic activities by competes with ATP for Hsp70 binding [47]. Hsp70 has been shown as a putative receptor, supplementary receptor or receptor-associated protein to facilitate Japanese encephalitis virus [48], enterovirus 71 [28], or infectious bronchitis virus [20] invasion. Also, Hsp70 plays a negative regulator role of MDA5-MAVS signaling pathway to inhibit innate immunity response in vitro [40]. However, the mechanisms of Hsp70-induced up-regulation of PEDV mRNA and protein synthesis during viral invasion and replication stages are unknown and consequently need to be further investigated. These data indicated that synthesis and activity of Hsp70 positively regulate PEDV mRNA synthesis and protein expression in vitro.

In this study, we showed that cold exposure could induct Hsp70 levels in vivo and in vitro. We found that synthesis and activity of Hsp70 were essential for PEDV mRNA synthesis and protein expression in vitro. This feature may help explain the seasonality of PED epidemics. Although Vero E6 and IPEC-J2 cells are the most common cell line used to study PEDV infection [49], it should be important to further verify some of the key findings from this study in vivo, such as the effects of cold exposure on PEDV pathogenesis and immunity. Also, further studies are needed to understand the detailed mechanism of Hsp70 mediated enhancement effects on PEDV infection in vivo and in vitro.

## 5. Conclusions

These data support a positive regulatory role for Hsp70 in the PEDV mRNA synthesis and protein expression and suggest that Hsp70 participates at the different steps of the viral life cycle, such as invasion or replication. It should be helpful for understanding the seasonality of PED epidemics and development of novel antiviral therapies in the future.

## Figures and Tables

**Table 1:** Primer sequences used for quantitative real-time PCR analysis.

Gene	Reference Sequence	Primer Sequences (5′-3′)
Hsp70 (porcine)	NM_001123127.1	Forward: CCAATGGCATCCTGAGTGTGACAG Reverse: ACGAACCATCCTCTCCACCTCTTC
Hsp70 (monkey)	XM_012436705.2	Forward: ACATCAGCCAGAACAAGCGA Reverse: AGTCGATGCCCTCAAACAGG
PEDV ORF3	GU372744	Forward: GCACTTATTGGCAGGCTTTGT Reverse: CCATTGAGAAAAGAAAGTGTCGTAG
β-actin (porcine)	XM_021086047.1	Forward: CACGCCATCCTGCGTCTGGA Reverse: AGCACCGTGTTGGCGTAGAG
β-actin (monkey)	XM_017530736.1	Forward: AGGCTCTCTTCCAACCTTCCTT Reverse: ACGTCGCACTTCATGATCGA
